# Non-celiac gluten sensitivity - why worry?

**DOI:** 10.1186/1741-7015-12-86

**Published:** 2014-05-23

**Authors:** Knut E A Lundin

**Affiliations:** 1 Department of Gastroenterology, Oslo University Hospital Rikshospitalet, N-0498 Oslo, Norway; 2 Centre for Immune Regulation, University of Oslo, N-0316 Oslo, Norway; 3 Faculty of Medicine, University of Oslo, N-0316 Oslo, Norway

**Keywords:** Celiac disease, Diagnosis, FODMAP, Gluten, Gluten-free diet, Irritable bowel syndrome, Multicenter study, Non-celiac gluten sensitivity

## Abstract

Wheat, once thought to be a critical ingredient in a healthy diet, has become a major threat, according to public opinion. The term non-celiac gluten sensitivity has been widely adopted to describe a clinical entity characterized by symptoms induced by gluten without the diagnostic criteria found in other gluten-related disorders. However, it has not been shown that gluten *per se* is involved, and it can be debated if the condition is a disease. Nevertheless, a large number of individuals go gluten-free, avoiding wheat, rye and barley, even without a defined medical cause. In a study in *BMC Medicine*, Volta and colleagues from Italy report on a large, multicenter attempt to enumerate the prevalence of non-celiac gluten sensitivity in secondary gastroenterology care. They found that approximately 3% of their more than 12,000 patients fulfilled their criteria for non-celiac gluten sensitivity. However, we are still challenged with finding stricter clinical criteria for the condition, developing a usable clinical approach for gluten challenge in these individuals, and understanding the pathogenesis of the condition.

Please see related article http://www.biomedcentral.com/1741-7015/12/85.

## Background

In the Western world, consumption of wheat has increased over the last half-century [1], as have the standard of living and life expectancy. Wheat is now more desirable than rice in large populations in China and India [2]. However, we now see a trend in Europe, Australia, New Zealand and the US to avoid dietary wheat. The numbers are uncertain, but prevalence up to 6% has been suggested. This trend is so pronounced that the consumption of wheat has declined [1], and it raises important questions. Are disease mechanisms involved? Is there any health benefit or risk from avoiding gluten? Is the trend solely due to public pressure? Could all those consumers be mistaken? Why adopt a strict, restrictive diet without a well-defined medical reason?

‘Gluten’ as a term is complicated. A gluten-free diet (GFD) is one without wheat, rye and barley. Gluten also refers to the glue-ish mass remaining after washing wheat flour with water. In addition, gluten describes the storage proteins found in cereals, those proteins that have well-known baking properties. The term ‘gluten-related disorders’ is also complicated [3,4]. At least three clinical entities are recognized: celiac disease (CD), wheat allergy, and non-celiac gluten sensitivity (NCGS). CD is a gluten-induced inflammatory disorder of the small intestinal mucosa, with typical serology comprising immunoglobulin (Ig) A antibodies to the enzyme tissue transglutaminase or deamidated gliadin peptides. Wheat allergy is an acute anaphylactic condition, with the presence of IgE to gluten. Lastly, NCGS is characterized by clinical signs induced by gluten, but without the same diagnostic criteria. Thus, NCGS is so far defined only by clinical terms - with all the problems we often see when clinicians lack strict criteria. The link to and comparison with irritable bowel syndrome is obvious. Irritable bowel is also diagnosed clinically, and can be treated by food restriction [5], but whether these entities really overlap remains to be seen.

### Non-celiac gluten sensitivity versus celiac disease

In a study published in *BMC Medicine*, Volta and colleagues report a multicenter, prospective study on prevalence of NCGS [6]. Thirty-seven specialist centers participated, all with expertise in gluten-related disorders. In Italy, such centers are accredited by the government and are responsible for reimbursement for GFD. The study evaluated 12,255 patients. The authors found 486 patients (3.19%) with NCGS, with a mean age 38 years, and a female to male ratio of 5.4:1. At the same time, they identified CD in 340 patients (2.77%). In the NCGS population, half of them had signs compatible with irritable bowel syndrome and received that diagnosis as well. Other frequent findings were allergies, autoimmune diseases and a close relative with CD. Laboratory tests showed low levels of ferritin, folic acid and vitamin D, but only in a minority. They found increased levels of IgG to gluten, but only in 25% of their patients. Disease controls were not reported. The authors should be congratulated for their efforts.

Although well conducted, there are some comments to be made. Firstly, the cut-off for diagnosis of NCGS in the study was not clear. The authors used a 60-item doctor-answered questionnaire with ‘yes/no’ items. There was no requirement for a given number of positive answers for a diagnosis. It appears that the diagnosis of NCGS was done ‘clinically’. Secondly, it is unclear if the participating clinicians in fact approved the patient’s use of their GFD. Thirdly, the study did not include a systematic placebo-controlled food challenge. This is often considered the gold standard for food intolerance, but is clinically problematic [7].

The study of Volta *et al*. showed NCGS in approximately 3% of referred patients. How this relates to the general population is unclear. Other reports have shown wide variations in prevalence. A study from a referral US center found NCGS in 6% of patients [4]. A US-based population survey study found a low prevalence of NCGS [8], but the survey was not designed to detect NCGS. A UK-based population study showed that 13% considered themselves as gluten-sensitive, and 3.7% consumed a GFD (most of them without a CD diagnosis) [9]. In New Zealand, 5% of children are on a GFD [10]. Thus, it is reasonable to accept that a noteworthy proportion of the Western population is on a GFD.

Are there reasons to worry about people having a GFD? We have no estimates on any health benefits of a GFD in the NCGS population, in contrast to the known benefits in the CD population. We do not yet have any proof that a GFD has a negative health effect on the NCGS population, whereas there is some evidence that it may in CD, because treated patients show signs of nutritional deficiency [11]. Thus, the short answer is that in the case of NCGS a GFD is neither medically needed nor detrimental. However, NCGS has many similar aspects to irritable bowel syndrome, a condition that has a severe impact on a patient’s quality of life and represents a very significant financial burden on all Western health care system. It is therefore necessary to developing a usable clinical approach for gluten challenge in individuals with NCGS.

Well, then, how to investigate? It depends on whether the patient is still on a gluten-containing diet or a GFD. The objective signs of CD, with serology and mucosal inflammation, normalize when a patient with CD commences a GFD. Thus, serological testing with anti-tissue transglutaminase IgA should be done before any clinician advocates a GFD. If the patient is already gluten-free, another approach should be taken. In fact, CD is not frequent in the NCGS population, and a clinical response to gluten withdrawal is not proof that the patient has CD [12-14]. Genetic analysis for human leukocyte antigens DQ2 and DQ8 is useful, because it is highly unlikely that CD is the causal disease in the absence of these genetic variants [15,16]. Further investigation of possible CD involves gluten challenge of two to six weeks’ length or more where the aim is to provide objective signs of CD [17,18].

Gluten challenge or withdrawal to prove or disprove NCGS is complicated. A blinded challenge approach would be ideal, but the standard test with flour in capsules is problematic and rarely used [7]. A good placebo food preparation is essential, and has been developed in Australia [19,20]. Further understanding of NCGS will require that other groups also introduce blinded, placebo-controlled challenge protocols. It has been questioned if NCGS actually exist, or if it is caused by sensitivity to fermentable carbohydrates that are abundant in gluten-containing cereals [20]. To further complicate the matter, non-gluten proteins have been proposed to be important [21], but for the wheat-sensitive patient, this does not really matter. Moreover, the use of a wide array of symptom evaluations and lack of a standardized symptom scoring form represent major obstacles to further progress.

## Conclusions

The use of a GFD without a well-defined medical reason has reached considerable proportions in many Western societies. The clinical entity NCGS resembles, and may well be a part of, irritable bowel syndrome. The diagnosis of both conditions is based on clinical work-up; objective biomarkers are not available and may be difficult to develop. NCGS may be caused by improper immune responses, intolerance to poorly digestible and fermentable substances in the wheat, or a combination of these. NCGS is a burden for the affected individuals and for the healthcare system trying to provide proper help for them. Future research should address whether gluten really is involved, and if a manageable, clinically acceptable, placebo-controlled investigation procedure can be developed. We should aim provide relief for those who really benefit from dietary restriction, without over-treating those who would benefit from more temporary interventions.

## Abbreviations

CD: celiac disease; GFD: gluten-free diet; Ig: immunoglobulin; NCGS: non-celiac gluten sensitivity.

## Competing interest

The author declares that he has no competing interests.
